# Effect of intermittent shade on nitrogen dynamics assessed by ^15^N trace isotopes, enzymatic activity and yield of *Brassica napus* L.

**DOI:** 10.3389/fpls.2022.1037632

**Published:** 2022-11-16

**Authors:** Hafiz Hassan Javed, Yue Hu, Muhammad Ahsan Asghar, Marian Brestic, Majid Ali Abbasi, Muhammad Hamzah Saleem, Xiao Peng, Abu Zar Ghafoor, Wen Ye, Jing Zhou, Xiang Guo, Yong-Cheng Wu

**Affiliations:** ^1^ College of Agronomy, Sichuan Agricultural University, Chengdu, China; ^2^ Key Laboratory of Crop Ecophysiology and Farming System in Southwest China, Chengdu, China; ^3^ Department of Biological Resources, Agricultural Institute, Centre for Agricultural Research, ELKH, Martonvásár, Hungary; ^4^ Department of Plant Physiology, Slovak University of Agriculture, Nitra, Slovakia; ^5^ Department of Biochemistry Ghulam Muhammad Mahar Medical College Sukkur, Shaheed Mohtarma Benazir Bhutto Medical University Larkana, Larkana, Pakistan; ^6^ College of Plant Science and Technology, Huazhong Agricultural University, Wuhan, China; ^7^ Sichuan Province Agro-meteorological Center, Chengdu, China

**Keywords:** nitrogen, ^15^N isotopes, carbohydrates, yield, shade, rapeseed

## Abstract

Modern era of agriculture is concerned with the environmental influence on crop growth and development. Shading is one of the crucial factors affecting crop growth considerably, which has been neglected over the years. Therefore, a two-year field experiment was aimed to investigate the effects of shading at flowering (S1) and pod development (S2) stages on nitrogen (N) dynamics, carbohydrates and yield of rapeseed. Two rapeseed genotypes (Chuannong and Zhongyouza) were selected to evaluate the effects of shading on ^15^N trace isotopes, enzymatic activities, dry matter, nitrogen and carbohydrate distribution and their relationship with yield. The results demonstrated that both shading treatments disturbed the nitrogen accumulation and transportation at the maturity stage. It was found that shading induced the downregulation of the N mobilizing enzymes (NR, NiR, GS, and GOGAT) in leaves and pods at both developmental stages. Shading at both growth stages resulted in reduced dry matter of both varieties but only S2 exhibited the decline in pod shell and seeds dry weight in both years. Besides this, carbohydrates distribution toward economic organs was declined by S2 treatment and its substantial impact was also experienced in seed weight and seeds number per pod which ultimately decreased the yield in both genotypes. We also revealed that yield is positively correlated with dry matter, nitrogen content and carbohydrates transportation. In contrast to Chuannong, the Zhongyouza genotype performed relatively better under shade stress. Overall, it was noticed that shading at pod developmental stage considerable affected the transportation of N and carbohydrates which led to reduced rapeseed yield as compared to shading at flowering stage. Our study provides basic theoretical support for the management techniques of rapeseed grown under low light regions and revealed the critical growth stage which can be negatively impacted by low light.

## 1 Introduction

In the modern era, agriculture is concerned with the environmental impact on crop yield and nutritional quality. Rapeseed (*Brassica napus* L.) is one of the most frequently consumed oilseeds crop worldwide, with double the oil yield per hectare as soybean. After rice, maize, and wheat, rapeseed is China’s fourth most farmed crop (Hu et al., 2022). The Yangtze River Basin is the main rapeseed-producing region, where farmers adopt an intensive cropping system to get better yields (Li et al., 2018). Furthermore, the demand for rapeseed oil as a sustainable energy source has risen significantly (Ahmad et al., 2011). Light is possibly the most geographically and temporally variable of all the environmental conditions that affect plant performance (Nascimento et al., 2015). Light signals photomorphogenesis and supplies energy to develop plant assimilatory power (Kumar et al., 2016). Global climate change has reduced daylight hours and solar radiation during the last 50 years (Ren, 2005). Clouds and greater plant populations can restrict light availability, especially in later growth phases. Under the influence of meteorological and environmental factors, the tallest crops are frequently susceptible to low light stress or self-shading (Gao et al., 2018). The impact of shade stress depends on the cultivar, growth stage, shading intensity, and shading duration. Shade stress damages the plant’s morphology and ultrastructure, limiting chlorophyll synthesis and lowering the canopy’s photosynthetic capability (Li et al., 2010; Mu et al., 2010; Bellasio and Griffiths, 2014). As a result, shading stress lowers photosynthate production and grain yield (Clay et al., 2009; Chikov et al., 2016; Ren et al., 2016). Light plays a vital role in plants’ photosynthate accumulation and nutrient intake and distribution (Clay et al., 2009; Cui et al., 2013). Absorption, assimilation, and transport of nitrogen (N) directly impact growth and development (Bu et al., 2014; Jia et al., 2014; Ihtisham et al., 2018). Nitrate is the most prevalent form of nitrogen available to plants due to the quick nitrification of the regularly used reduced forms of nitrogen. After being absorbed by the plant, nitrate needs to be converted to an ammoniacal form in order to be incorporated into amino acids for protein synthesis. The first enzyme that carries out the rate-limiting step in converting nitrate to ammonia in the nitrate assimilatory pathway is nitrate reductase, which is substrate-inducible (Eilrich and Hageman, 1973). Inorganic nitrogen can only be absorbed and utilized when transformed into organic nitrogen, with glutamate and glutamine being the major assimilation metabolites generated from ammonia. Glutamine synthetase (GS)/glutamate synthase (GOGAT) was discovered to catalyze ammonia assimilation (Lea and Miflin, 1974) and it was determined to be the principal mechanism for ammonia assimilation in higher plants (Hirel et al., 2001; Miflin and Habash, 2002; Glevarec et al., 2004; Martin et al., 2006). The transamination that transfers amino groups from glutamate to other amino acids perform crucial functions in nitrogen metabolism (Lea et al., 1992). The accumulation and partition of photosynthate determine the grain yield (Sun et al., 2017; Zhai et al., 2017). Before anthesis, a large number of carbohydrates and nitrogenous chemicals accumulate, which are then reallocated to the grain (Yang et al., 2001; Xu et al., 2006). The content of grain N depends on the rate of nitrogen accumulation and proportion of translocation from distinct organs of crop (Chen et al., 2015a). Additionally, the ratio of nitrogen translocated from the vegetative organs to the grain is influenced by climatic factors, management techniques, soil nutrients, and water availability, which are crucial for crop yield (Dordas and Sioulas, 2009). Yangtze river basin is the part of southern region of China. As a result of the significantly decreased light intensity in southern China, where plants face low light stress during different growth stages of different crops (Setién et al., 2013; Gao et al., 2017b). Thus, it is critical to explore the accumulation and remobilization of dry matter (DM), N and carbohydrates under shading stress at different growth stages of rapeseed. Although many studies have examined the changes in N distribution in response to various growth conditions such as temperature, precipitation, and nitrogen-deposition conditions (Villar-Salvador et al., 2015), but very few have focused on the effects of shade stress on N assimilation and distribution at different growth stages of crops especially the rapeseed. So, we hypothesized that low light stress at pod development stage significantly altered the N dynamic, carbohydrates transportation and ultimately causes the yield reduction. Artificial shade environments were used to simulate the field shade conditions to investigate the plant dry matter and nitrogen accumulation processes. The accumulation and translocation of nitrogen were investigated using the ^15^N stable isotope tracer under shading at various growth stages of rapeseed. The specific objectives of this study were to quantify the effects of various shading periods on rapeseed dry matter and N accumulation and to identify the critical growth stage that has the most significant impact on N dynamics and yield in rapeseed plants.

## 2 Materials and methods

### 2.1 Experimental location

**Figure 1:**
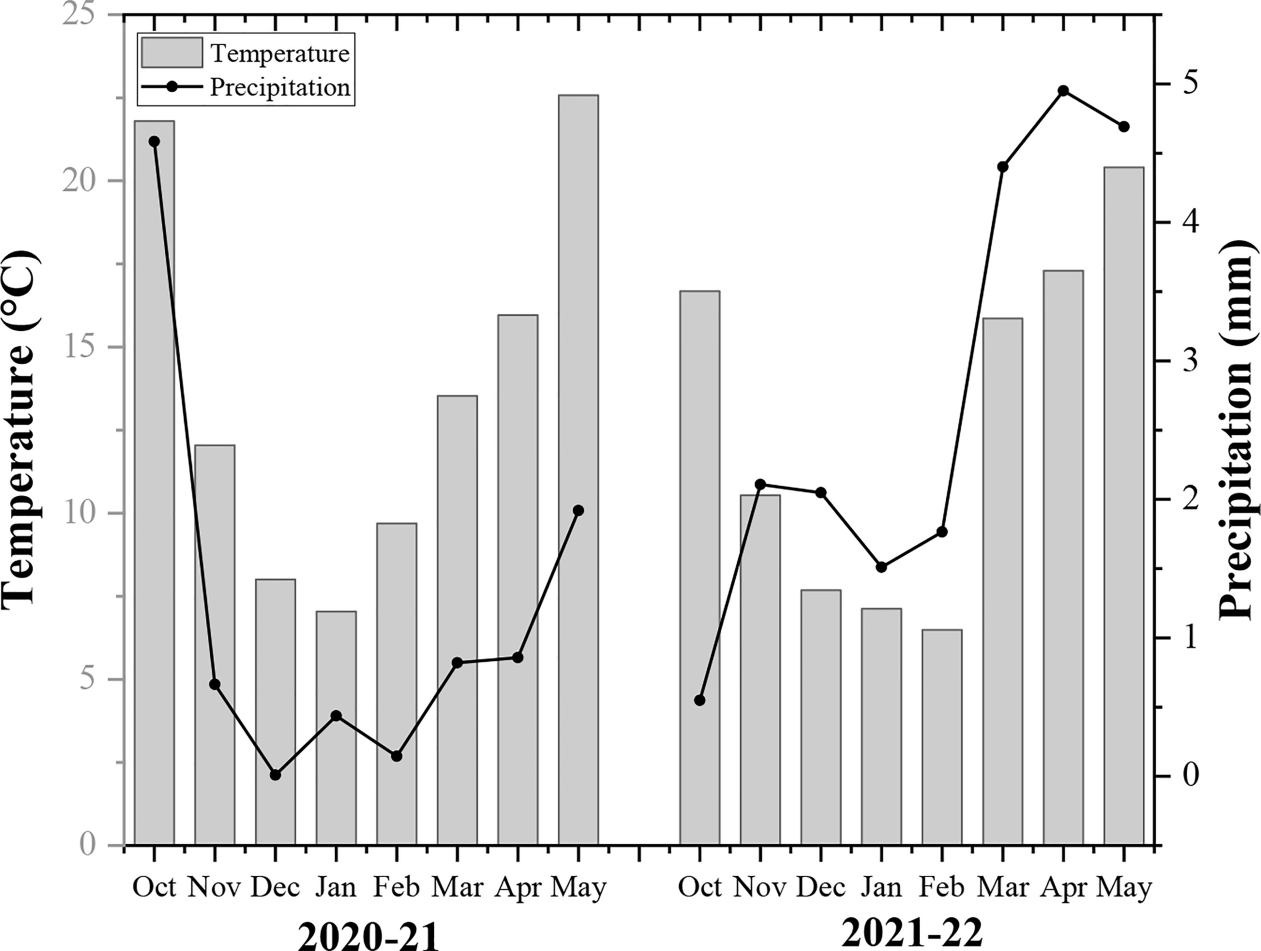
The monthly average temperature and precipitation of rapeseed growing seasons (2020-22).

A two-year field experiment was carried out at Huihe village, Chengdu plain, Sichuan province (102°54-104°53 E, 30°05-31°26 N) from 2020-22. It is a subtropical region with an average temperature of 16.1°C, annual total precipitation of 1780 mm, and a total sunshine duration of 1050 h (Sichuan Province Agro-meteorological Center, China). The basic soil fertility of soil includes organic matter (20.3 g/kg), total nitrogen (1.3 g/kg), available phosphorus (0.015 mg/kg), available potassium (0.118 mg/kg), and pH (6.7) in the topsoil layer (0-20cm). The monthly annual temperature and rainfall of the rapeseed growing season are demonstrated in **Figure 1**.

### 2.2 Experimental materials and layout

**Figure 2:**
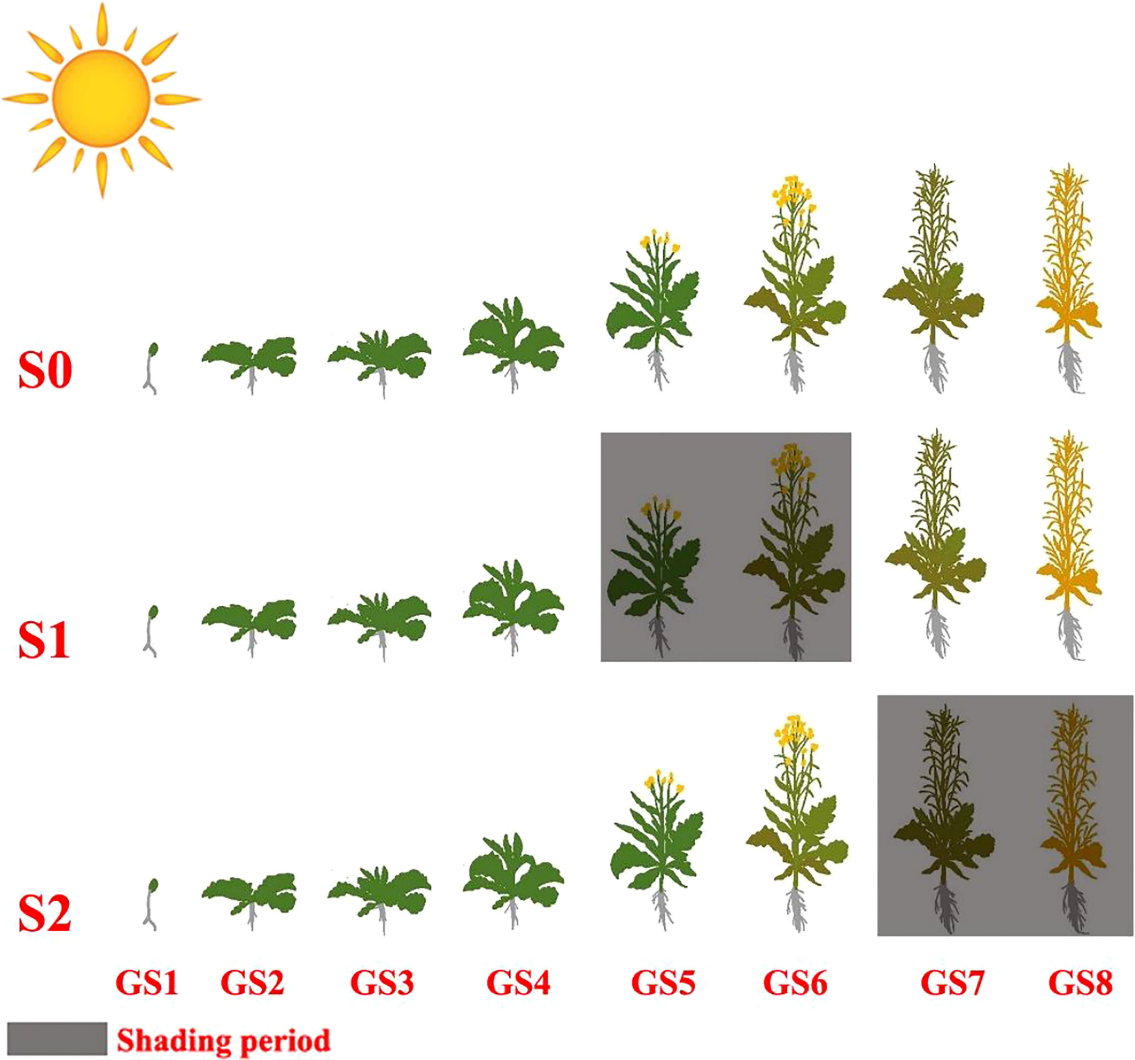
Diagrammatic representation of shading treatment at different growth stages of rapeseed. Control (ambient light) (S0); shade from GS5 to GS6 (S1); shade from GS7 to GS8 (S2); germination and emergence stage (GS1); leaf development stage (GS2); side-shoot development stage (GS3); stem prolongation stage (GS4); inflorescence emergence (GS5); flowering stage (GS6); pod development (GS7); harvesting stage (GS8).

The two rapeseed genotypes (Chuannong and Zhongyouza) were involved in the two-year field trial. These rapeseed genotypes are abundantly cultivated in Sichuan province, especially in the higher reaches of the Yangtze river basin. The experiment employed a two-factor split-plot design. Three shading treatments were established at various growth stages of rapeseed; S0 = control (ambient light), S1 = shade from GS5 to GS6 and S2 = shade from GS7 to GS8 (**Figure 2**). The plants were enclosed by a layer of black polyethylene nets, which blocked approximately 35% of solar radiation. Two cultivars were assigned to the main plot and subplots received shading treatment. All treatments were carried out three times, yielding 24 plots with a 12 m^2^ plot size. The prior harvested crop was rice, and the soil fertility was medium. The field was rotated before sowing, and a rope was manually pulled on-line while maintaining a row-row distance of 33 cm and a plant-plant gap of 20 cm. One seedling was left in each hole after emergence, and the baseline planting density was 150,000 plants/ha. Phosphorus and potassium fertilizers were applied at a rate of 90 kg/ha as base fertilizer. Nitrogen fertilizer was used at a 90 kg/ha rate in split dosages of 50% as base fertilizer + 50% topdressing at seedling stage. Local measurements were practiced to control the pests and weeds.

### 2.3 Sampling and measurement

#### 2.3.1 Yield parameters

At maturity, 10 plants were chosen to measure the number of pods per plant, number of seeds per pod, 1000 seed weight and yield of both genotypes.

#### 2.3.2 Dry matter determination

At maturity stage, 10 plants were separated into stems, pod shells, and seeds to determine the dry matter. After that, samples were dried for 30 minutes at 105°C, followed by drying at 80°C, until a consistent weight was attained and the data was recorded as dry matter.

#### 2.3.3 Determination of nitrogen content

The 6 plants were divided into stems, leaves, pod shells, and seeds at the GS6 and GS8 stages. Afterward, the samples were dried at 105°C for 30 minutes to stop the enzymatic activity, then dried at 80°C till a constant weight was obtained. Samples were then mashed with a mortar and sieved through a 0.5 mm sieve. The semi-automatic Kjeldahl nitrogen analyzer (FOSS 2300) was used to calculate total nitrogen content (Sparks et al., 2020). The following indices were calculated by following the previously published methods (Dordas and Sioulas, 2009; Gao et al., 2020):


$${\text{NT (g plant}}^{-1})=N at GS6-N at GS8\left(\text{stem }+\text{pod shell }+\text{leaf (vegetative components})\right)$$

NTE (%) = (NT/N content at GS6)×100

NCP (%) = (NT/seed N at GS8)×100

$${\text{NHI (g plant}}^{-1})\;=\text{ Seed N at GS}8/\text{total N of above-ground biomass at GS}8$$

$${\text{NA (g plant}}^{-1})=\text{Seed N at GS}8/\text{NT}$$

Note: N=nitrogen; GS6=pod development growth stage; GS8=harvesting stage; NT=nitrogen translocation; NTE= nitrogen translocation efficiency; NCP=nitrogen contribution proportion; NHI=nitrogen harvest index and NA=nitrogen assimilation.

The plants (3) with similar phenological characteristics of each plot were labeled with ^15^N at GS5. Labeled plants of each plot were harvested at the end of GS7 and divided into leaves, stem, pod shell and grain. The samples were dried at 105°C for 30 minutes and then at 80°C in an oven (DHG-9423A Shanghai SANFA Scientific Instrument Co., Ltd.) to attain a constant weight. All of the samples were grounded into powder and sieved at 200 mesh. The enrichment of 15N in 4 mg powdered plant samples was determined using an isotope 100 mass spectrometer (Isoprime, Manchester, UK). The control treatment was measured based on the plants without ^15^N isotopes tracing. The accumulation of ^15^N in organs was calculated as follows (Clay et al., 2016):


$${\;}^{15}\text{N accumulation of plant organ}=\text{dry matter weight}\times \text{N concentration}\times^{15}\text{N enrichment}$$

#### 2.3.4 Assay of NR, NiR, GS and GOGAT activities

Samples of fresh leaves and pods were collected in liquid nitrogen at 10-day intervals following shading to assess enzyme activity. According to previously described procedures, the enzymes nitrate reductase (NR), nitrite reductase (NiR), glutamine synthetase (GS), and glutamate synthase (GOGAT) were examined (LIANG et al., 2011; Majláth et al., 2016; Khan et al., 2020).

#### 2.3.5 Total non-structural carbohydrates

The plant samples were oven-dried for 30 minutes at 105°C before being kept at 80°C till they reached a consistent weight. After that, samples were pulverized in an electric mortar and 0.1 g of powder was added to 6 mL of 80% ethanol, which was then put in water bath at 80°C for 40 minutes and centrifuged for 5 minutes at 5000 rpm. The supernatant was transferred to 50 mL tubes as the main solution, and the procedure was repeated twice. To make the primary solution 50 mL, 80% ethanol was added. For decolorization, 0.1 g charcoal solution was added to the primary solution, and the primary solution was filtered to use for the following analyses (Asghar et al., 2020).

##### 2.3.5.1 Determination of sucrose

To determine sucrose content, 0.9 mL of primary solution was taken into test tubes and 0.1 mL of 2 M NaOH was added and placed in the water bath for 10 minutes. After heating, samples were allowed to cool at room temperature for 15 minutes. After that mixture was heated at 80°C with 3 mL of 10 M HCL and 1 mL of 0.1% resorcinol for 10 minutes. The supernatant was taken and absorbance was measured in a spectrophotometer at 480 nm (Spectra Max i3x from Austria) (Ghafoor et al., 2021).

##### 2.3.5.2 Determination of reducing sugar

In 10 mL test tubes, 1.5 mL primary solution, 0.5 mL deionized H_2_O, and 1.5 mL DNS solution were mixed to determine the reducing sugars. After that, the tubes were placed in 80°C water bath for 10 minutes. A spectrophotometer measured the absorbance at 520 nm in the supernatant (Spectra Max i3x from Austria).

##### 2.3.5.3 Determination of soluble sugar

To measure the soluble sugar, 20 mL of test tubes were filled with 1 mL of primary solution and 4 ml of 0.2% sulfate anthrone combination. After that, samples were heated for 15 minutes in a water bath and cooled for 15 minutes at room temperature. The supernatant was measured at 480 nm in a spectrophotometer (Spectra Max i3x from Austria) (Raza et al., 2021).

### 2.4 Statistical analysis

The data was recorded and sorted out by Microsoft Excel 2019. SPSS 19.0 (SPSS, Chicago, IL, USA) software was used to statistically analyze all the data. To estimate the differences among treatments, ANOVA with three-way analysis of variance followed by least significant difference (LSD) at *p*<0.05 significance level was performed. Pearson correlation coefficient were calculated to determine the relationship between different parameters. All the tables and figures were shaped by Excel 2019 and Origin 2021 software (OriginLab Co., Northampton, MA, USA).

## 3 Results

### 3.1 Effect of shade on the yield attributes of rapeseed genotypes

**Figure 3:**
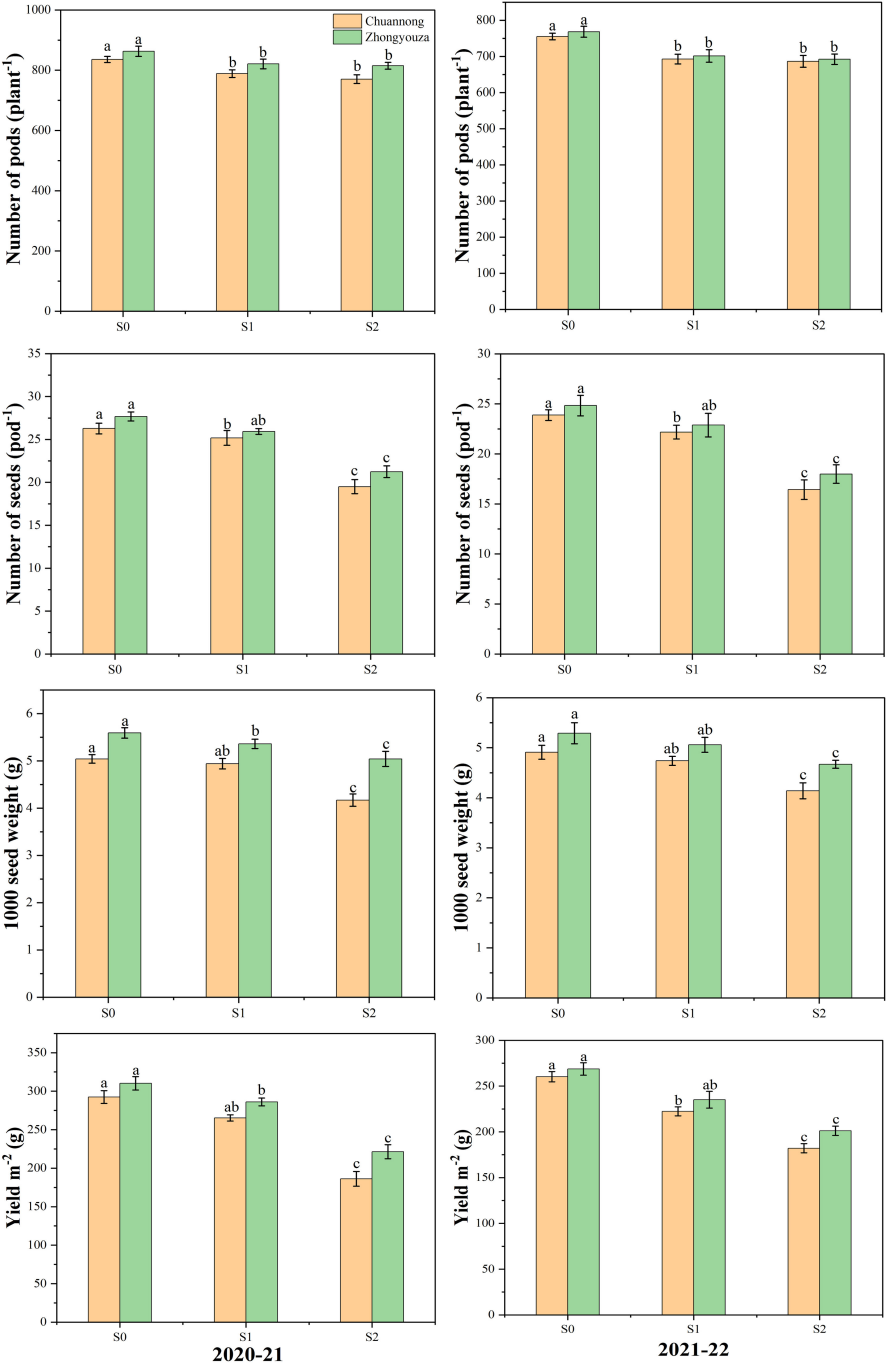
Effect of shading on yield parameters of rapeseed. S0= control (ambient light); S1= shade at the whole flowering stage and S2= shade at the start of pod development to pod maturity. Values were determined using the (n=10) LSD test, and various small letters denote the significance level of treatments at the 0.05 probability level (Duncan test).

Different shading treatments significantly altered the yield variables of both investigated rapeseed genotypes. In contrast to S0, the Chuannong genotype showed a decreased number of pods by 7.40 and 9.23% and Zhongyouza genotype experienced the 7.16 and 8.25% after S1 and S2 treatments as compared to S0, respectively. While the number of seeds per pod was lowered by 5.91 and 39.60% in Chuannong and 7.58 and 33.85% in Zhongyouza after the respective shading treatments. Under S1 and S2, the Chuannong exhibited 2.78 and 19.73% reduction and Zhongyouza showed 4.42 and 12.04% decline in 1000-seed weight following S1 and S2, respectively. In case of yield, the S1 and S2 declined the yield of Chuannong genotype by 13.31 and 50.03% and this reduction was 11.06 and 37.01% in Zhongyouza, respectively. Under various shading treatments, the aforementioned yield characteristics in both years demonstrated a similar trend, while 2020-21 year significantly exhibited higher yield in both genotypes (**Figure 3**). Additionally, S2 had a significant impact on all yield parameters. Taken altogether, it was observed that the Chuannong genotype was more shade-sensitive and showed lower yield than Zhongyouza under shade treatment.

### 3.2 Impact of shade stress on dry matter accumulation of rapeseed

Shade stress significantly reduced the dry matter of both rapeseed genotypes. According to two-year average data, the DM was reduced by 7.28 and 33.32% in Chuannong and 7.16 and 31.91% in Zhongyouza following S1 and S2 treatment as compared to S0, respectively. Shading at both growth stages disrupted the accumulation and distribution of DM in the organs of rapeseed. Under S2, a significant drop of DM was detected in the rapeseed organs. The seed weight was more affected by shading than stem and pod shells at the organ level under S2.

**Figure 4:**
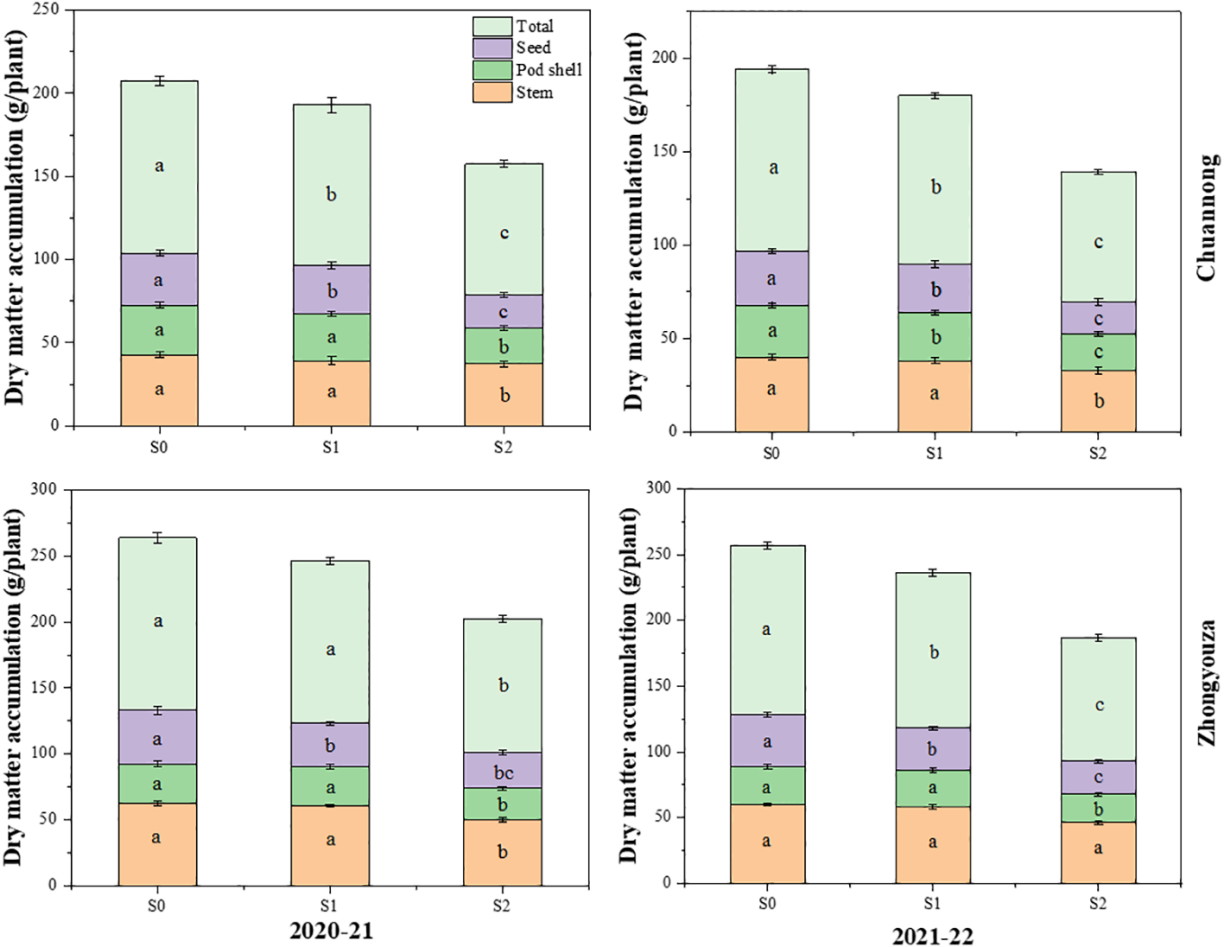
Effect of shading on dry matter accumulation in rapeseed. S0= control (ambient light); S1= shade at the whole flowering stage and S2= shade at the start of pod development to pod maturity. Values were determined using the (n=10) LSD test, and various small letters denote the significance level of treatments at the 0.05 probability level (Duncan test).

Contrary to S0, the Chuannong genotype showed the 8.96 and 58.34% decline in seed weights after S1 and S2 treatments as compared to S0, respectively, while Zhongyouza exhibited 22.9 and 49.63% inhibition after the respective shading treatments. The stem, pod shell, and seed weights of both genotypes under shading followed a similar decreasing trend: S2<S1<S0 (**Figure 4**). The DM of all organs followed the same reducing tendency in both years. but 2020-21 displayed higher dry matter than 2021-22 year. Aside from that, shade during the pod stage (S2) substantially impacted both cultivars’ dry matter.

### 3.3 Shade-dependent changes in nitrogen accumulation and distribution in rapeseed

The differences in nitrogen accumulation and distribution were found under shading stress at distinct growth stages. The values in **Tables 1** and **2** represent the mean value for two-year experiment. The total nitrogen (TN) of both genotypes was detected in the following increasing order: S0>S1>S2 at maturity stage. In contrast to S0, S1 and S2 treatments reduced the TN distribution of Chuannong by 17.84 and 73.29%, respectively, however this reduction was 8.47 and 40.27% in Zhongyouza, respectively (**Table 1**). Shading had an impact on rapeseed organs of both genotypes. For instance, S1 had lower nitrogen values for leaves and stems, whereas pod shells and seeds showed lower nitrogen values under S2. Regarding genotypes, a higher TN was observed in Zhongyouza. Moreover, shading treatments affected both genotypes’ N contents of the leaves, stems, and pods (**Table 1**).

**Table 1:** Effect of shading on accumulation and distribution of nitrogen in rapeseed.

		N accumulation at pod development (g plant^˗1^)				N distribution at maturity (g plant^˗1^)			
Varieties	Treatments	Leaves	Stem	Pod	Total	Stem	Pod shell	Seed	Total
Chuannong	S0	2.02 ± 0.17b	1.23 ± 0.06a	2.13 ± 0.09a	5.39 ± 0.16b	1.74 ± 0.11b	1.08 ± 0.08a	5.35 ± 0.01b	8.18 ± 0.10b
	S1	1.13 ± 0.08c	0.26 ± 0.11c	1.33 ± 0.13b	2.72 ± 0.06de	0.48 ± 0.02d	1.02 ± 0.05a	5.04 ± 0.02c	6.54 ± 0.06d
	S2	2.29 ± 0.19ab	1.25 ± 0.22a	2.06 ± 0.03a	5.60 ± 0.03b	1.59 ± 0.15b	0.56 ± 0.02d	3.13 ± 0.03d	5.28 ± 0.10f
Zhongyouza	S0	2.55 ± 0.13a	1.42 ± 0.11a	2.10 ± 0.09a	6.08 ± 0.03a	1.95 ± 0.02a	0.89 ± 0.03b	6.14 ± 0.01a	8.97 ± 0.01a
	S1	1.09 ± 0.02c	0.56 ± 0.03bc	1.47 ± 0.03b	3.13 ± 0.01c	1.05 ± 0.03c	0.73 ± 0.01c	5.06 ± 0.01c	6.83 ± 0.03c
	S2	2.53 ± 0.14b	1.39 ± 0.06a	2.14 ± 0.04a	6.05 ± 0.16a	1.83 ± 0.02a	0.63 ± 0.03cd	3.12 ± 0.03d	5.57 ± 0.02e
Variance analysis	Y	**	**	**	*	**	**	**	**
	V	**	**	ns	**	**	**	**	**
	T	**	**	**	*	**	**	**	**
	Y**×**V	ns	ns	ns	ns	ns	ns	ns	ns
	Y**×**T	ns	ns	ns	ns	ns	ns	ns	ns
	V**×**T	*	ns	ns	*	**	**	**	**
	**Y×**V**×**T	ns	ns	ns	ns	ns	ns	ns	ns

S0, control (ambient light); S1, shade at the whole flowering stage and S2, shade at the start of pod development to pod maturity. Values were determined using the (n=6) LSD test, and various small letters denote the significance level of treatments at the 0.05 probability level (Duncan test). Y, V and T represent the year, variety and treatment, while **, * and ns denote the highly significant, significant and non-significant.

The lower value of N translocation (NT), N translocation efficiency (NTE) and N contribution proportion (NCP) was perceived in S1, whereas higher values were examined in S2 treatment. The NTE was 5.30 and 36.78% lower in Chuannong and 23.08 and 37.08% in Zhongyouza genotype under S1 as compared to S0 and S2, respectively. The N harvest index (NHI) and N assimilation (NA) values of both genotypes were lowest in S2 treatment (**Table 2**).

**Table 2:** Effect of shading on nitrogen translocation (NT), nitrogen translocation efficiency (NTE), nitrogen contribution proportion (NCP), nitrogen harvest index (NHI) and nitrogen assimilation (NA) in rapeseed.

Varieties	Treatments	NT(g plant ^˗1^)	NTE(%)	NCP(%)	NHI (%)	NA (g plant ^˗1^)
Chuannong	S0	2.56 ± 0.18d	47.44 ± 2.32c	47.84 ± 3.36b	0.65 ± 0.01d	2.79 ± 0.17b
	S1	1.22 ± 0.01e	45.05 ± 0.74c	24.28 ± 0.22c	0.77 ± 0.01a	3.81 ± 0.02a
	S2	3.45 ± 0.18ab	61.62 ± 3.04a	110.38 ± 5.92a	0.59 ± 0.03f	0.12 ± 0.18d
Zhongyouza	S0	3.24 ± 0.04c	53.27 ± 0.57b	52.78 ± 0.79b	0.68 ± 0.01c	2.89 ± 0.05b
	S1	1.35 ± 0.04e	43.28 ± 1.09c	26.75 ± 0.74c	0.74 ± 0.01b	3.70 ± 0.04a
	S2	3.59 ± 0.07a	59.33 ± 0.37a	115.30 ± 3.06a	0.56 ± 0.01g	0.18 ± 0.09d
Variance analysis	Y	**	**	**	**	**
	V	**	ns	*	*	ns
	T	**	**	**	**	**
	Y**×**V	ns	ns	ns	ns	ns
	Y**×**T	ns	ns	ns	ns	ns
	V**×**T	**	**	ns	**	ns
	**Y×**V**×**T	ns	ns	ns	ns	ns

S0, control (ambient light); S1, shade at the whole flowering stage and S2, shade at the start of pod development to pod maturity. Values were determined using the (n=6) LSD test, and various small letters denote the significance level of treatments at the 0.05 probability level (Duncan test). Y, V and T represent the year, variety and treatment. While **, * and ns denote the highly significant, significant and non-significant.

**Figure 5:**
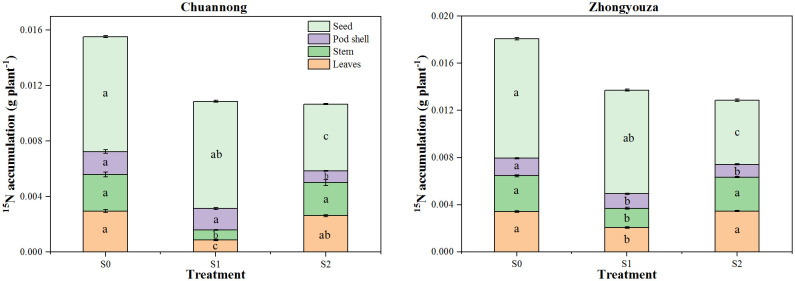
Distribution of ^15^N to different plant organs of rapeseed at maturity under different shade conditions. S0= control (ambient light); S1= shade at the whole flowering stage and S2= shade at the start of pod development to pod maturity. Values were determined using the (n=3) LSD test, and various small letters denote the significance level of treatments at the 0.05 probability level (Duncan test).

Shading decreased the distribution of ^15^N isotopes in different organs of rapeseed (**Figure 5**). Compared to S0, the stem ^15^N accumulation was declined in Chuannong genotype by 69.31 and 12.85% under S1 and S2, respectively, however, this change was 54.14 and 5.12% in Zhongyouza genotype. In Chuannong, the reduction of 7.17 and 72.35% in seeds ^15^N accumulation was observed following S1and S2 relative to S0, respectively. While this inhibition was 15.31 and 86.38% for Zhongyouza. Leaf and stem ^15^N accumulation of both genotypes displayed a increasing trend; S0>S2>S1. While pod shell and seeds exhibited a increasing trend; S0>S1>S2. The ^15^N accumulation in the entire plant decreased under both shade tratements, compared to S0. In general, both rapeseed genotypes exhibited the following ^15^N accumulation trend; S0>S1>S2. Taken altogether, it was noticed that the Zhongyouza displayed a higher accumulation of ^15^N than Chuannong (**Figure 5**).

### 3.4 Shade-induced modifications in enzymatic activities of rapeseed

**Figure 6:**
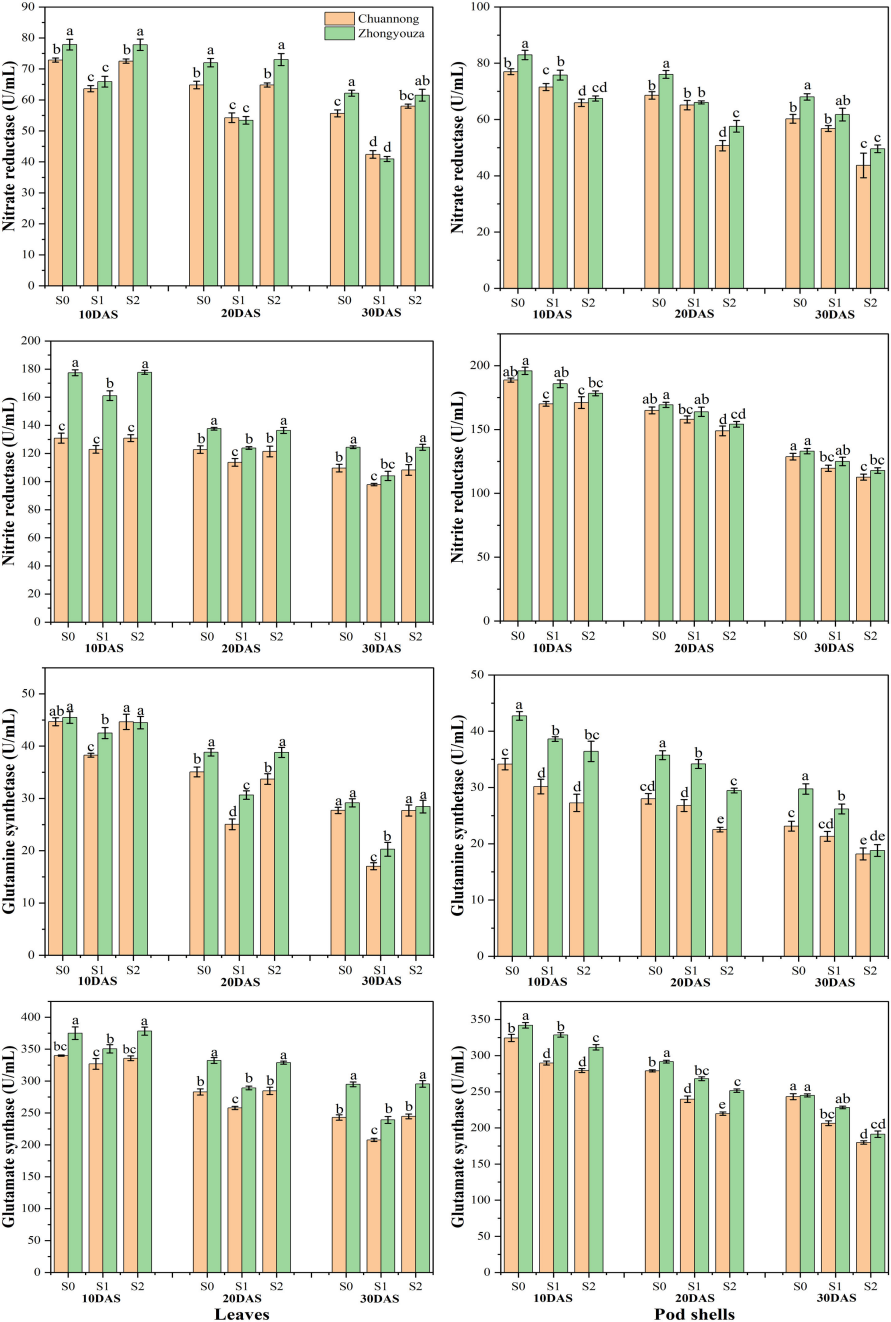
N mobilizing enzymatic activities under shade stress. S0= control (ambient light); S1= shade at the whole flowering stage and S2= shade at the start of pod development to pod maturity. Values were determined using the LSD test, and various small letters denote the significance level of treatments at the 0.05 probability level (Duncan test).

The shading stress at both growth stages considerably influenced the enzymatic activities in the leaves and pod shells of both the studied genotypes. Relative to S0, the S1 reduced the NR, NiR, GS and GOGAT activities by 20.65, 8.60, 33.74 and 9.24% in leaves of Chuannong, respectively. Whereas the Zhongyouza experienced a 28.31, 12.96, 21.47 and 14.05% reduction following S1 as compared to S0, respectively (**Figure 6**).

In case of pod shell, the NR activity of Chuannong was reduced by 6.37 and 28.33% after S1 and S2 relative to S0, respectively, while this decline was 11.51 and 30% for Zhongyouza genotype. A decline of 7.75 and 11.47% was detected in NiR activity of Chuannong and 4.98 and 10.56% Zhongyouza genotypes under S1 and S2 when compared with S0, respectively. The S1 and S2 treatments also declined the pod shell GS activity of Chuannong (8.91 and 25.45%) and Zhongyouza (9.31 and 27.74%), respectively. Similarly, the pod shell GOGAT activity showed 15.05 and 24.67% decline in Chuannong and 6.50 and 16.46% in Zhongyouza genotype following S1 and S2, respectively (**Figure 6**). Comparing both genotypes, our findings unveiled that the Chuannong cultivar showed higher NR and GOGAT activity while Zhongyouza showed more NiR and GS enzymatic activities under S1 and S2 treatments. Furthermore, comparing S1 and S2, S2 significantly lowered all the enzymatic activities in both genotypes.

### 3.5 Shade-mediated modifications in carbohydrates accumulation at maturity

The changing trend of carbohydrates content of the both rapeseed genotypes was the same in both years. The shading treatment considerably reduced the sucrose, reducing sugar and soluble sugar contents of the stem and pod shell of both tested genotypes. The sucrose content of stem was declined by 14.08 and 41.28% in Chuannong and 6.87 and 35.36% in Zhongyouza, while pod shell showed a reduction of 16.45 and 35.18% in Chuannong and 14.19 and 37.99% in Zhongyouza following S1 and S2 treatments as compared to S0, respectively (average value based on two years). Generally, it was noticed that Zhongyouza genotype showed higher sucrose content than Chuannong genotype under all treatments.

Under various shading treatments, the reducing sugar content of Chuannong and Zhongyouza showed the following trend: S0>S1>S2 in both years. Compared with S0, the stem reducing sugar contents of Chuannong genotype experienced a decline by 15.21 and 76.66%, while this reduction for Zhongyouza genotype 10.71 and 51.21% after S1 and S2 treatments, respectively. In addition, the S1 and S2 decreased the reducing sugar of pod shell by 25.53 and 84.37% in Chuannong genotype and 15.52 and 55.81% in Zhongyouza genotype, respectively.

The soluble sugar content of Zhongyouza genotype was higher than that of Chuannong under all the treatments. Contrary to control, the stem soluble sugar of Chuannong genotype was inhibited by 10.52 and 46.72% and Zhongyouza genotype was reduced by 10 and 44.26% following S1 and S2 treatments, respectively. However, pod shell soluble sugar content showed a decline of 8.56 and 36.24% in Chuannong and 8.21 and 34.39% in Zhongyouza genotypes after the respective shading treatments. Moreover, the following inclination of carbohydrates was observed in both cultivars; S0>S1>S2 (**Table 3**). Furthermore, Zhongyouza showed significantly higher carbohydrates content in stem and pod shell and 2020-21 year showed higher values of carbohydrates as compared to 2021-22. Collectively, it was seen that the shade at the pod development stage (S2) significantly affected the carbohydrates content in both years.

**Table 3:** Effect of shading on carbohydrates content of rapeseed at maturity.

			Stem carbohydrates at maturity (mg/g)			Pod shell carbohydrates at maturity (mg/g)		
Years	Varieties	Treatments	Sucrose	Reducing sugar	Soluble sugar	Sucrose	Reducing sugar	Soluble sugar
2020-21	Chuannong	S0	4.86 ± 0.02b	0.53 ± 0.01b	3.36 ± 0.03b	5.38 ± 0.01b	0.59 ± 0.01b	4.06 ± 0.05b
		S1	4.26 ± 0.03c	0.46 ± 0.01c	3.04 ± 0.03d	4.62 ± 0.04d	0.46 ± 0.02c	3.74 ± 0.04d
		S2	3.44 ± 0.09e	0.30 ± 0.01e	2.29 ± 0.01f	3.98 ± 0.03f	0.32 ± 0.01e	2.99 ± 0.02f
	Zhongyouza	S0	5.13 ± 0.04a	0.63 ± 0.01a	3.52 ± 0.01a	5.63 ± 0.02a	0.66 ± 0.01a	4.22 ± 0.02a
		S1	4.80 ± 0.03b	0.56 ± 0.01b	3.20 ± 0.01c	4.93 ± 0.02c	0.58 ± 0.01b	3.90 ± 0.01c
		S2	3.80 ± 0.03d	0.41 ± 0.01d	2.44 ± 0.02e	4.08 ± 0.05e	0.43 ± 0.01cd	3.14 ± 0.03e
								
2021-22	Chuannong	S0	4.1 ± 0.02b	0.49 ± 0.01b	3.05 ± 0.03b	4.61 ± 0.01b	0.52 ± 0.01b	3.74 ± 0.03b
		S1	3.50 ± 0.03c	0.42 ± 0.01c	2.73 ± 0.03d	3.85 ± 0.04d	0.39 ± 0.02c	3.42 ± 0.03d
		S2	2.67 ± 0.08e	0.26 ± 0.01e	1.97 ± 0.01f	3.20 ± 0.03f	0.25 ± 0.01e	2.67 ± 0.01f
	Zhongyouza	S0	4.37 ± 0.04a	0.58 ± 0.01a	3.21 ± 0.01a	4.86 ± 0.02a	0.59 ± 0.01a	3.90 ± 0.01a
		S1	4.04 ± 0.03b	0.51 ± 0.01b	2.89 ± 0.01c	4.16 ± 0.02c	0.51 ± 0.01b	3.58 ± 0.01c
		S2	3.04 ± 0.03d	0.37 ± 0.01d	2.13 ± 0.02e	3.31 ± 0.05e	0.36 ± 0.01d	2.82 ± 0.02e
Variance analysis		Y	*	**	*	*	**	*
		V	**	**	**	**	**	**
		T	*	**	*	*	**	*
		Y**×**V	ns	ns	ns	ns	ns	ns
		Y**×**T	ns	ns	ns	ns	ns	ns
		V**×**T	**	*	ns	**	*	ns
		**Y×**V**×**T	ns	ns	ns	ns	ns	ns

S0, control (ambient light); S1, shade at the whole flowering stage and S2, shade at the start of pod development to pod maturity. Values were determined using the (n=10) LSD test, and various small letters denote the significance level of treatments at the 0.05 probability level (Duncan test). Y, V and T represent the year, variety and treatment. While **, * and ns denote the highly significant, significant and non-significant.

### 3.6 Correlation analysis

**Figure 7:**
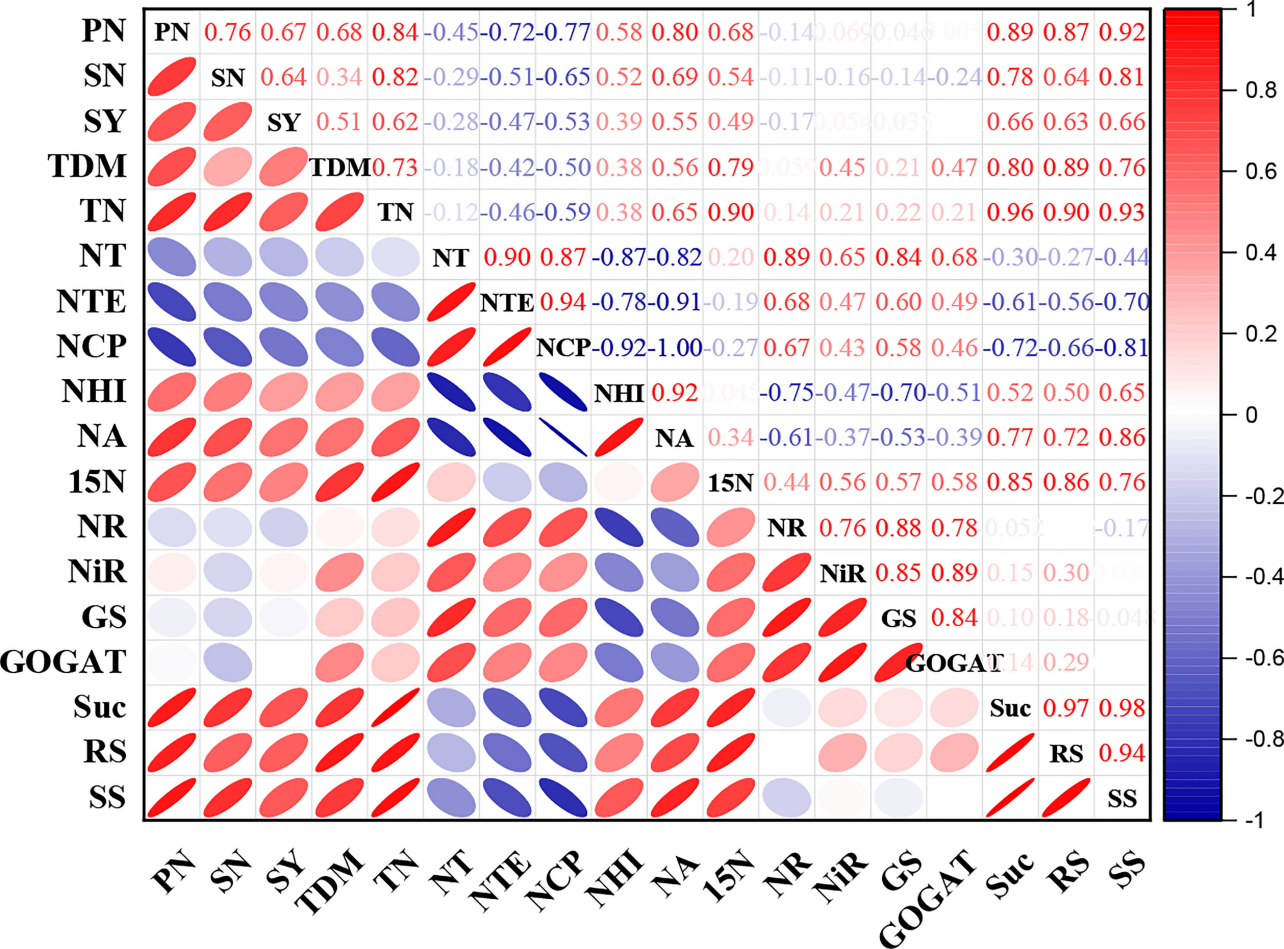
Correlation analysis between agronomic traits, nitrogen content, carbohydrates and yield. Red and blue color represents the positive and negative correlation. The size and intensity of color exhibited the significance of variables. PN, pod number; SN, seed number; SY, seed yield; TDM, total dry matter, TN, total nitrogen; NT, nitrogen translocation, NTE, nitrogen translocation efficiency; NCP, nitrogen contribution proportion; NHI, nitrogen harvest index; NA, nitrogen assimilation; 15N, ^15^nitrogen isotope; NR, nitrate reductase; NiR, nitrite reductase; GS, glutamine synthetase; GOGAT, glutamate synthase; Suc, sucrose; RS, reducing sugar and SS, soluble sugar.

The current study’s correlation analysis demonstrated that shade stress was substantially connected to yield metrics, nitrogen absorption and carbohydrates transportation. All the enzyme activities were significantly positive correlated with N transportation to different organs but a non-significant correlation of enzymatic activities with yield was observed. A negative correlation of NT, NTE, NCP and NA with carbohydrates were examined but carbohydrates exhibited positive correlation with yield parameters. Moreover, total dry matter and ^15^N displayed a significantly positive correlation with seed yield (**Figure 7**).

## 4 Discussion

### 4.1 Response of yield parameters and dry matter under shade stress

Light is a critical environmental component impacting the growth and development of crops (Guoping et al., 2008; Zhong et al., 2014). Numerous studies have documented a decrease in yield due to shading stress (Cantagallo et al., 2004; Zhang et al., 2006; Acreche et al., 2009; Mu et al., 2010). In two-year studies, there were no significant declines in pods per plant but a significant fall in pod filling and grain weight (Wang et al., 2015). Previous researches have demonstrated that the drop in grain production was due to a reduction in grain number and weight (Acreche et al., 2009; Mu et al., 2010; Polthanee et al., 2011). Variations in ovule fertility and seed number per pod were impacted by changes in growth circumstances such as N availability, light and temperature (Bouttier and Morgan, 1992). Grain yield and spikelet filling had significant positive linear associations, while grain yield and grain weight showed a positive relationship (Wang et al., 2015). Shading reduced grain dry weight during grain filling, lowering grain yield (Ishibashi et al., 2014). To pinpoint crucial growth stage, most other reported field experiments have not used sufficiently defined durations of shading. For instance, (Habekotté, 1993) and (Iglesias and Miralles, 2014) both used shade (60% and 50%, respectively) for entire anthesis stage and resulting in yield losses of 50% and 15%, respectively, but no particular growth stage could be determined. Thus, to our knowledge, our study is among the fewer studies of rapeseed that has identified a relatively critical growth stage which affected by shading.

We found that shade in the beginning of the pod’s development limited the assimilates transfer and decreased the weight of the pod shell and the number of seeds per pod (Tayo and Morgan, 1979). In the current study, the S1 demonstrated a relatively high yield when the supply of pods and seeds resumes to normal levels after the shade has been removed, although a shortage of assimilates on flowers under shade is also damaging and diminishes the potential for compensatory growth. As previously discussed, I canola appears more vulnerable to severe temperatures and water deficits during late blooming and early pod set, aligning its sensitive periods with those of pulses rather than cereals (Sadras and Dreccer, 2015). Thus, they function similarly to the shade treatments applied in this study. In general, the observed correlations between the time of shade treatments and their effects on yield components and their relationships at maturity are similar to previously described physiological consequences of reduced assimilate supply (Tayo and Morgan, 1979; Keiller and Morgan, 1988; Diepenbrock, 2000). Dry matter production and accumulation are the primary determinants of crop yield, which are also limited by different environmental factors (CH, 1995). Shading stress greatly changed the physiology and morphology of the plant and eventually decreased the dry matter accumulation and distribution, resulting in decreased grain yield (Acreche et al., 2009; Li et al., 2010; Mauro et al., 2011). Dry matter accumulation in high-yield maize accounts for more than 60% of the total dry matter. Grain yield is influenced by the development and distribution of dry matter in vegetative organs such as the stem, leaf, and sheath (Huang et al., 2007). Our results demonstrated that S2 treatment considerably reduced the pod shell and seeds dry weight, which caused the yield drop in both rapeseed genotypes. We can conclude that shade at pod development stage (S2) is crucial to cause reduction in dry matter and yield.

### 4.2 N accumulation and distribution under shading conditions

**Figure 8:**
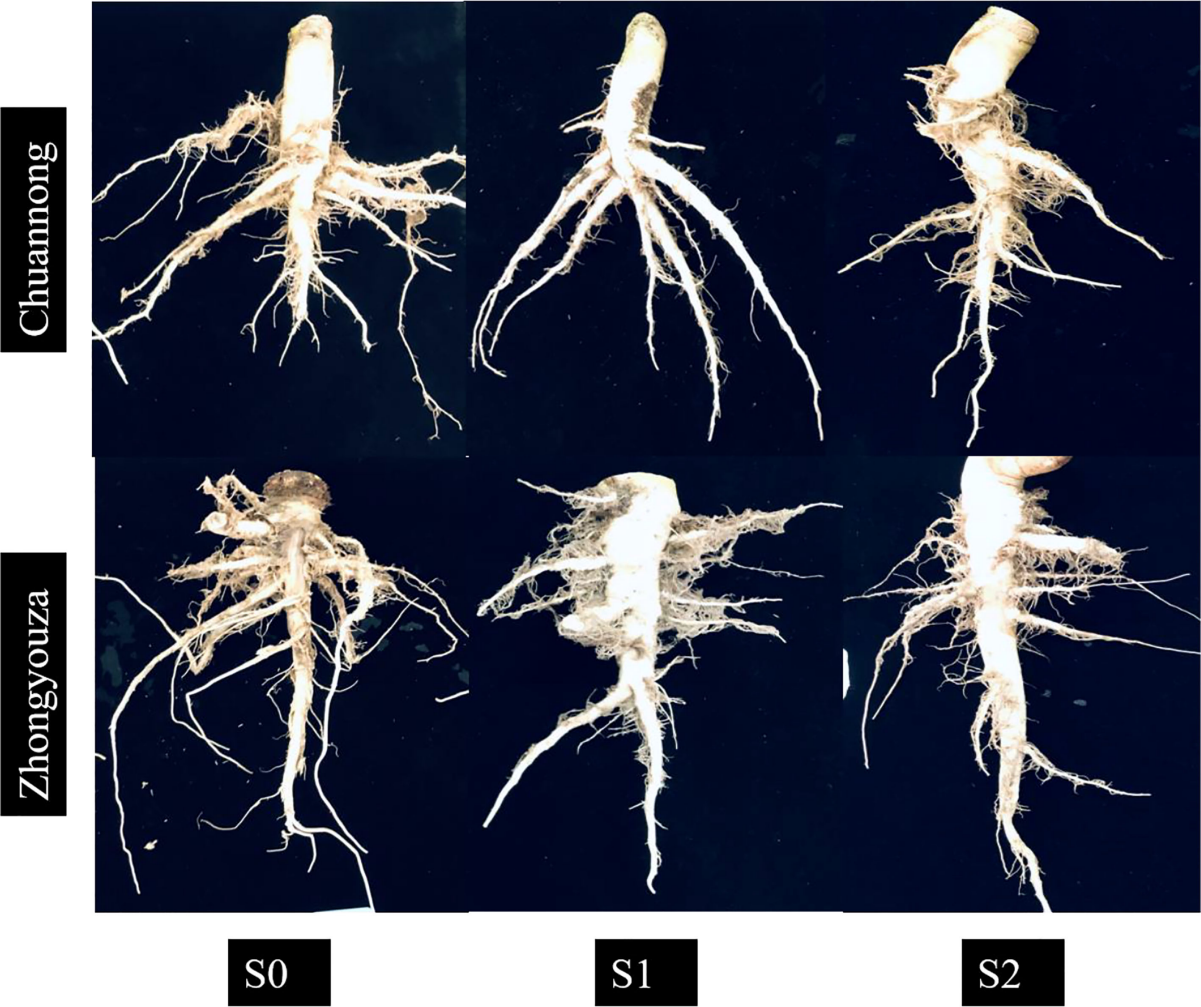
Effect of shading stress on the roots structure of two rapeseed genotypes.

Increasing biological yield is the foundation for increasing output; nutrient intake and distribution are key prerequisites for biological yield (Hirel et al., 2007). This study examined the changes in N accumulation and transportation under shade at various growth stages and deduced a portion of the mechanism underlying the grain yield response to N use. The remobilization of nitrogen in vegetative organs and the uptake of additional nitrogen throughout the grain-filling cycle provide grain N (Mueller and Vyn, 2016). Furthermore, nitrogen remobilization in the stem and leaf accounts for 69 to 80% of grain N (Subedi and Ma, 2005; Chen et al., 2014b). As a result, N accumulation and distribution in vegetative and reproductive organs play a pivotal role in dry matter weight at maturity and influence grain yield. According to our findings, shade declined the total N accumulation of rapeseed in the following order: S0>S1>S2>. The total N of the pod shell and seeds were significantly reduced by shade at pod development (S2) compared to the flowering stage (S1). Furthermore, N buildup of S1 raised after the light was restored, but it did not return to normal levels (**Table 1**; **Figure 5**). The S2 inhibited the amount of N translocation towards pod shell and seeds compared to other treatments (**Table 1**), resulting in poorer yields (Chen et al., 2015b). When the accumulated N at pod development is smaller than the grain requirements, nitrogen transport rises (Chen et al., 2015b), as evidenced by our findings. Late-season shade (S2) reduced the N translocation towards economic organs (**Table 1**). As a result, we found that shading reduced N uptake distribution in all organs, resulting in a decrease in seed yield. In conclusion, shade reduced N buildup and impeded N transfer from vegetative organs to grain, such as leaves, stems, and pod shells. This study found that shading at pod developmental stage (S2) had a greater detrimental impact on N uptake than at flowering stage (S1), consistent with root shape and root physiology changes during shading (**Figure 8**) (Gao et al., 2017a). Shading altered the root structure, reducing root dry weight, absorption area, and active absorption area. Weather, climate, and air pollution contribute to shade, which is a challenging problem to solve in the manufacturing process. Changing sowing times is an excellent way to deal with low-light prone areas at later growth stage of crop, but it can be effected by temperature, soil moisture, and crop rotation as well (Gao et al., 2017a; Zhao et al., 2018).

### 4.3 Shade-dependent changes in N metabolizing enzyme activities

The leaf N content and enzyme activities are closely associated with each other (Sinclair et al., 2000). We observed that, shade inhibited the activity of NR, NiR, GS, and GOGAT, as was shown by the earlier research (Wang et al., 2020). GS and GOGAT are two essential enzymes involved in the N metabolism (Nigro et al., 2017). Due to shade, GS and GOGAT activities reduced gradually in the present study. This observation in grains was the same as in leaves (Wang et al., 2020). Wheat responds as a sensitive to ammonium nutrition at low light intensities, and its low GS activity is insufficient for ammonium assimilation. This occurrence apparently arose as a result of the significantly decreased light intensity in southern China, where plants face weak light stress during grain filling, which is relatable to our findings (Setién et al., 2013; Gao et al., 2017b). When plants were subjected to shade, nitrate delivery to the tops dropped considerably. The drop in NR activity resulted from the reduction in nitrate concentration (Udayakumar et al., 1981). Reduced NR activities in shade-adapted plants make it easier for the plants to coordinate their N and carbon uptake across a variety of light conditions (Fredeen et al., 1991). The 50.3, 24 and 30.4% inhibition was also observed in NR, GS and GOGAT enzymatic activities following shade treatment (Yu et al., 2011). We concluded that leaves and pods’ enzymatic activities are greatly reduced by shade. Among the shading treatments, the S2 treatment considerably declined the enzyme activities in the pods, which restricted the nitrogen transport towards seeds that led to low grain yield in both the investigated rapeseed genotypes.

### 4.4 Carbohydrates accumulation and distribution under shade

Shading limited the transformation of photosynthetic products. It accelerates the consumption of assimilates in leaves and stems and reduces grain yield (Li et al., 2013). Studies on different crops showed that the carbohydrate accumulation in leaves, stems, and roots decreased significantly under shading (Chen et al., 2014a; Hussain et al., 2021). As one of the main photosynthetic products, sucrose is significantly affected by light intensity and light cycle (Emerson, 1958). The decrease in sucrose content is closely related to light intensity. Shade reduce the output of leaves, the primary organ responsible for the formation of photosynthetic products, and eventually results in a decrease in sucrose content (Wu et al., 2017). The deleterious effect of whole-plant shade during grain filling on grain yield has been ascribed to photo-assimilate deficiency (Singh and Jenner, 1984; Okawa et al., 2003). However, there were differences in the accumulation and transport of carbohydrates under shading stress at different growth stages. This study showed that shading at pod stage (S2) had a more serious impact than shading at flowering stage (S1), which directly led to the reduction in grain yield. This could be because of leaf senescence, reducing photosynthetic potential, carbon fixation, and assimilates at pod development stage (Brouwer et al., 2012), which resulted in insufficient transportation of photosynthetic products. It was discovered that shaded wheat reduced grain output by speeding up the consumption of assimilates in the leaves and stems (Li et al., 2013). The authors further found that the carbohydrate of pod photosynthesis is mostly transported to the grain. In maize plants, the post-anthesis shading weakened the ability of nitrogen accumulation and stimulated the obvious re mobilization of carbohydrate reserves from stem to grain, but the decrease of grain filling rate eventually led to the decrease of grain yield (Reed et al., 1988). In this study, shading at flowering stage still reduced grain yield. The retardation can be attributed to the loss of non-structural carbohydrate transport to the kernel as a result of light deprivation (Mu et al., 2009) and kernel filling rate (Jichao and Zhiyong, 2005), which decreased the endosperm cell number and volume (Jia et al., 2011) and kernel set as a result of accelerated senescence. Starch deposition was decreased by shading, particularly under high shading. Additionally, ear shading decreased the kernel starch (Cui et al., 2012). Based on our findings, we can conclude that S2 treatment significantly reduced the carbohydrates translocation towards economic organs, leading to lower yields in both rapeseed genotypes.

## 5 Conclusion

Shading stress decreased DM and N accumulation, N transportation and distribution in multiple organs of rapeseed genotypes, and decreased the grain N content, which consequently reduced yield. The leaf and pod enzyme activities were also considerably influenced by the shade stress, which are associated with N accumulation and distribution. Relative to flowering stage, the shading at pod development stage significantly inhibited the carbohydrates transportation towards seeds. The Zhongyouza genotype outperformed Chuannong in all the aforementioned parameters under shade stress. Based on our findings, the current study provides the deeper insights into the effect of shade stress on the physio-biochemical mechanisms of rapeseed genotypes, which could be helpful for the management techniques of rapeseed grown under low light regions.

## Data availability statement

The original contributions presented in the study are included in the article/Supplementary Material. Further inquiries can be directed to the corresponding authors.

## Author contributions

Conceptualization: Y-CW and HJ. Methodology: HJ, YH and WY. Data collection: HJ, JZ, XG, YH. Formal analysis and investigation: HJ, YH, JZ and XP. Writing - original draft preparation: HJ and MuAA. Writing - review and editing: HJ, MuAA, AG. Supervision: Y-CW. All authors contributed to the article and approved the submitted version.

## Funding

This research was funded by Sichuan Province Crop Breeding Research and Cultivation Project (2021YFYZ0005), Biological breeding major science and technology project of Sichuan Province (2022ZDZX0015) and National Modern Agricultural Industrial Technology System Sichuan Rapeseed Innovation Team (sccxtd2022-03).

## Conflict of interest

The authors declare that the research was conducted in the absence of any commercial or financial relationships that could be construed as a potential conflict of interest.

## Publisher’s note

All claims expressed in this article are solely those of the authors and do not necessarily represent those of their affiliated organizations, or those of the publisher, the editors and the reviewers. Any product that may be evaluated in this article, or claim that may be made by its manufacturer, is not guaranteed or endorsed by the publisher.
